# Epigenetic Mechanisms Underlying Developmental Plasticity in Horned Beetles

**DOI:** 10.1155/2012/576303

**Published:** 2012-03-05

**Authors:** Sophie Valena, Armin P. Moczek

**Affiliations:** Department of Biology, Indiana University, 915 E Third Street, Myers Hall 150, Bloomington, IN 47405-7107, USA

## Abstract

All developmental plasticity arises through epigenetic mechanisms. In this paper we focus on the nature, origins, and consequences of these mechanisms with a focus on horned beetles, an emerging model system in evolutionary developmental genetics. Specifically, we introduce the biological significance of developmental plasticity and summarize the most important facets of horned beetle biology. We then compare and contrast the epigenetic regulation of plasticity in horned beetles to that of other organisms and discuss how epigenetic mechanisms have facilitated innovation and diversification within and among taxa. We close by highlighting opportunities for future studies on the epigenetic regulation of plastic development in these and other organisms.

## 1. Introduction

Organismal form and function emerge during ontogeny through complex interactions between gene products, environmental conditions, and ontogenetic processes [1, 2]. The causes, nature, and consequences of these interactions are the central foci of epigenetics [3]. Broadly, epigenetics seeks to understand how phenotypes emerge through developmental processes, and how that emergence is altered to enable evolutionary modification, radiation, and innovation. Epigenetic mechanisms can operate at any level of biological organization above the sequence level, from the differential methylation of genes to the somatic selection of synaptic connections and the integration of tissue types during organogenesis. Here, we take this inclusive definition of epigenetics and apply it to the phenomenon of developmental plasticity, defined as a genotype's or individual's ability to respond to changes in environmental conditions through changes in its phenotypes [4]. All developmental plasticity is, by definition, epigenetic in origin, as the genotype of the responding individual remains unaltered in the process. It is the nature, origins, and consequences of the underlying epigenetic mechanisms that we focus on in this review. We do so with specific reference to horned beetles, an emerging model system in evo-devo in general and the evolutionary developmental genetics of plasticity in particular.

 We begin our review with a general introduction to the concept of developmental plasticity. We then introduce our focal organisms, horned beetles, summarize the most relevant forms of plasticity that have evolved in these remarkable organisms, review what is known about the underlying epigenetic mechanisms, and highlight future research directions. Lastly, we discuss how studies in *Onthophagus* species could provide meaningful insight into three major foci in evo-devo research: the development and evolution of shape, the process of evolution via genetic accommodation, and the origin of novel traits. We begin, however, with a brief introduction of the significance of plasticity in development and evolution.

## 2. The Biology of Developmental Plasticity

Developmental plasticity refers to an individual's ability to respond to environmental changes by adjusting aspects of its phenotype, often in an adaptive manner. In each case a single genotype is able, through the agency of environment-sensitive development, to give rise to vastly different phenotypes. Developmental plasticity is perhaps most obvious in the expression of alternative morphs or polyphenisms, as in the seasonal morphs of butterflies, winged or wingless adult aphids, aquatic or terrestrial salamanders, or the different castes of social insects (reviewed in [2]). However, developmental plasticity is also inherent in more modest, often continuous changes in response to environmental conditions, such as tanning (in response to sun exposure), muscle buildup (in response to workouts) or immunity (following an infection resulting in an immune response). Lastly, developmental plasticity is a necessary prerequisite for developmental canalization, or the production of an *in*variant phenotype in the face of environmental fluctuation. Here, plastic compensatory adjustments on some level of biological organization enable the homeostatic maintenance of developmental outputs at others, such as the maintenance of blood sugar levels in the face of fluctuating nutrition and activity, or the maintenance of scaling relationships despite nutrition-dependent variation of overall body size in most organisms. Developmental plasticity is thus a ubiquitous feature of organismal development, applicable to all levels of biological organization, and rich in underlying mechanisms.

 Developmental plasticity not only enables coordinated and integrated responses in development but also has great potential to affect evolutionary processes and outcomes (reviewed in [4, 5]). Developmental plasticity enables organisms to adaptively adjust their phenotype to changing environmental conditions. On one side, developmental plasticity may thus impede genetic divergences that might otherwise evolve between populations subject to disparate environmental conditions. On the other, plasticity buffers populations against local extinctions, thus increasing the opportunity for the evolution of local adaptations and diversification.

 Similarly, developmental plasticity may both impede and facilitate evolutionary diversification by providing additional targets for selection to operate on, by offering modules for the regulation of development that can be reused across developmental contexts, and by creating novel trait interactions. In each case, developmental plasticity may result in pleiotropic constraints on adaptive evolution, but also has the potential to shift the evolutionary trajectories available to lineages into phenotypic space that otherwise would remain unexplored [5].

 The role of developmental plasticity in evolution is perhaps most important when we consider the consequences of organisms encountering novel environments, for instance during the natural colonization of a new habitat or the anthropogenic alteration of ecosystems due to global climate change, habitat degradation, and the invasion of alien species [5]. Here, developmental plasticity enables the production of functional, integrated phenotypes, despite development occurring in previously unencountered, or greatly altered, conditions. Moreover, such novel conditions may result in the formation of novel traits or trait variants previously unexpressed, alongside the release of previously cryptic, conditionally neutral genetic variation. Developmental plasticity thus has the potential to determine which phenotypic and genetic variants become visible to selection in a novel environment, thus delineating the nature and magnitude of possible evolutionary responses. Consistent with a long-assumed role of developmental plasticity in evolution (reviewed in [2]), a growing number of artificial selection experiments on a broad range of organisms (*Drosophila*: [6]; but see [7], Arabidopsis [8], fungi [9], and Lepidoptera [10]) have now demonstrated unequivocally that developmental systems confronted with challenging or novel environments can indeed expose novel phenotypic and genetic variants that, in turn, provide ample substrate for rapid, selective evolution of novel phenotypes. Similarly, studies on natural populations are providing growing evidence that ancestral patterns of plasticity have enabled and guided more refined evolutionary responses in derived populations (e.g., [11]).

 Developmental plasticity thus plays a central role in the production and evolution of phenotypic variation. Further understanding of the nature of this role likely requires a thorough understanding of the epigenetic mechanisms that enable plastic responses to environmental variation. As outlined in the following sections horned beetles have begun to provide diverse opportunities to investigate the mechanisms underlying the epigenetic regulation of developmental plasticity and to probe their significance in the developmental origin and evolutionary diversification of form and behavior. We begin with a brief introduction of the biology of these organisms.

## 3. The Biology of Horned Beetles

**Figure 1:**
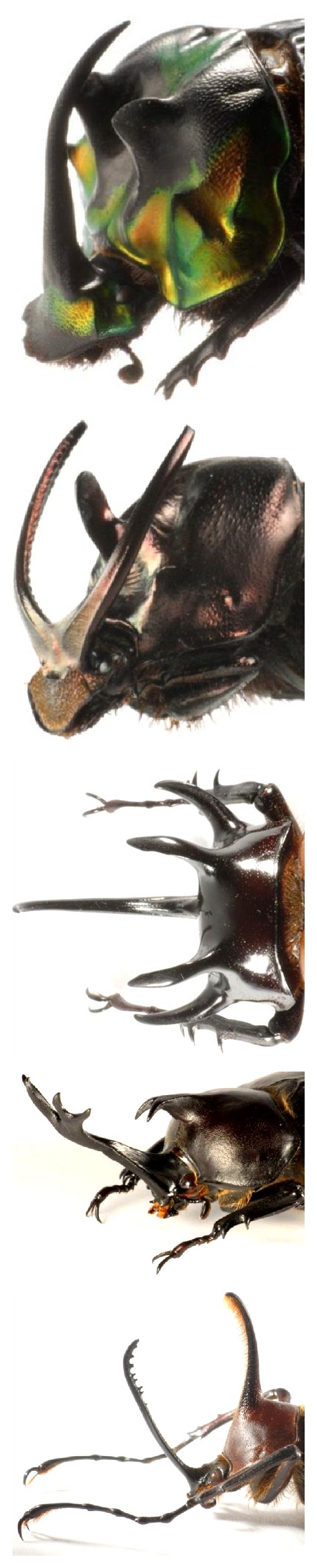
Examples of the exuberance and diversity of horn phenotypes across genera. top to bottom: Scarabaeinae:* Phanaeus imperator, Onthophagus watanabei; *Dynastinae:* Eupatorus gracilicornis, Trypoxylus (Allomyrina) dichotoma, Golofa claviger. *

Beetles are holometabolous insects and constitute the most diverse insect order on the planet. Horned beetles comprise a polyphyletic group of diverse beetle families marked by the development of horns or horn-like structures in at least some species (reviewed in [12, 13]). Horn evolution has reached its extremes, both in terms of exaggeration and diversity, in two subfamilies within the Scarabaeidae, the Dynastinae (i.e., rhinoceros beetles), and the Scarabaeinae, or true dung beetles (Figure 1). In both subfamilies, thousands of species express horns and have diversified with respect to location, shape, and number of horns expressed. In extreme cases, horn expression more than doubles body length and may account for approximately 30% of body mass.

 Despite the remarkable morphological diversity that exists among horned beetle species, horns are used invariably for very similar purposes: as weapons in aggressive encounters with conspecifics (reviewed in [12]). In the vast majority of species, horn expression is restricted to, or greatly exaggerated in, males, and absent or rudimentary in females. In these cases horns are used by males as weapons in male combat over access to females (e.g., [14]). In all species studied to date, body size has emerged as the most significant determinant of fighting success. In a subset of species, horns are expressed by both sexes. Here, males and females use horns as weapons in defense of mates and nesting opportunities, respectively (e.g., [15]). Lastly, in a very small number of species, horn expression is exaggerated in females and greatly reduced in males. Such reversed sexual dimorphisms are rare and the ecological conditions that have facilitated their evolution are largely unknown [16, 17].

 We know most about the biology of horned beetles through studies on one particular genus in the Scarabaeinae: *Onthophagus. *Adults of the *Onthophagus* genus colonize dung pads of a variety of dung types, consume the liquid portions and bury the more fibrous fraction in subterranean tunnels as food provisions for offspring in the form of brood balls. Brood balls typically contain a single egg and constitute the sole amount of food available to a developing larva. Variation in the quantity or quality of parental provisions or abiotic factors such as soil moisture can greatly affect the amount of food that is effectively available to sustain larval development, which in turn results in substantial variation in larval mass at pupation and final adult body size, as detailed below.

 Also similar to many other horned beetles species, *Onthophagus* frequently have to contend with high levels of male-male competition for females and female-female competition over breeding resources such as dung and tunneling space [18]. This unique combination of developmental conditions (marked by partly unpredictable larval resources) and ecological conditions (marked by intense intraspecific competition) has facilitated the evolution of a remarkable degree of plasticity in development, physiology, and behavior in *Onthophagus* beetles, as overviewed in the next section.

## 4. Developmental Plasticity in Onthophagus

### 4.1. Plasticity in Timing of Life History Transitions

Larval *Onthophagus* develop in a partly unpredictable resource environment, as their feeding conditions depend on the quantity and quality of dung provisioned for them by their parents and the physical properties of the nesting site. Unlike the highly mobile larval stages of many other holometabolous insects, larval *Onthophagus* cannot change their location or add to the resources made available to them. *Onthophagus* larvae meet these unpredictable conditions with a striking degree of plasticity in the timing of life history transitions, specifically by molting to the pupal stage at a range of larval body sizes far greater than what has been observed for other insects (reviewed in [19]). For instance, *Onthophagus taurus* larvae will routinely feed for 15 days during the third and final larval instar under *ad libitum *conditions, but are capable of completing metamorphosis if food deprived after just 5 days of feeding. The resulting larvae pupate at a fraction of the body mass of larvae fed *ad libitum* and eclose as tiny adults. Such striking flexibility in the dynamics of larval development and the body mass at pupation allows *Onthophagus* larvae to respond to unpredictable variation in larval feeding conditions while ensuring eclosion to a viable adult capable of reproducing. As a consequence of this phenomenon, natural populations of adult *Onthophagus* commonly display a remarkable amount of intraspecific variation in male and female body sizes.

### 4.2. Morphological Plasticity

Recall that in the vast majority of species horn expression is restricted to males, which use horns in male combat over access to females or nesting sites. Recall also that body size is the most important determinant of fighting success, yet ecological conditions generate males of a wide range of body sizes, many of which are too small to succeed in aggressive encounters. In many horned beetle species, these conditions have led to the evolution of alternative male phenotypes, with large males relying on the use of horns and aggressive fights to secure mating opportunities, while smaller males rely on nonaggressive sneaking behaviors (discussed in detail below). *Morphologically*, male polyphenism has a range of manifestations.

**Figure 2:**
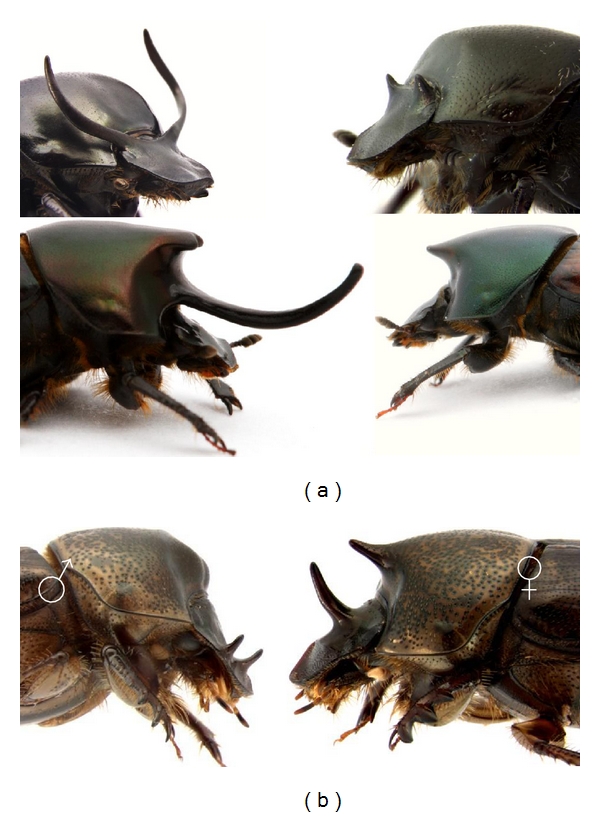
(a) Examples of male polyphenism in *O. taurus *(top) and *O. nigriventris *(bottom). Large males are shown on the left and small males on the right. Note that females (not shown) are entirely hornless in both species. (b) Rare reversed sexual dimorphism in *O. sagittarius*. Males also lost ancestral male dimorphism.

**Figure 3:**
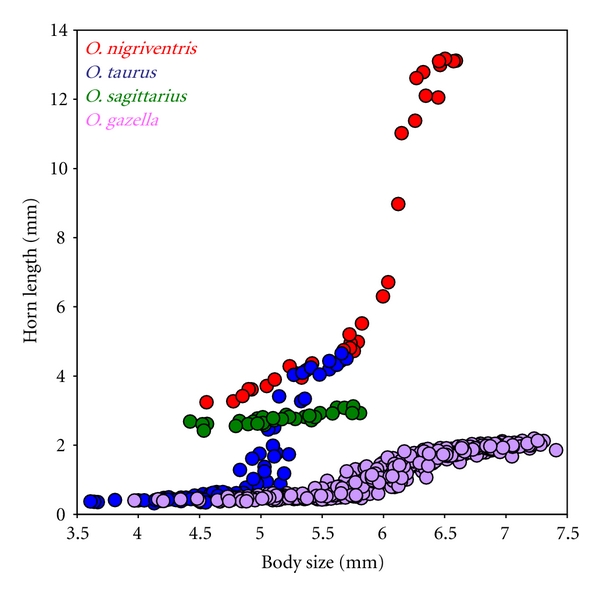
Differences among four *Onthophagus *species in the range of nutrition-mediated plasticity in male horn expression. Shown are the scaling relationships between body size (*X*-axis) and horn length (*Y*-axis). Patterns of nutritional plasticity in horn expression range from minimal and linear (*O. sagittarius*) and modestly sigmoidal (*O. gazella*) to strongly sigmoidal with species-specific differences in amplitude (*O. taurus* and *O. nigriventris*).

First, in numerous species horn expression is restricted to, or greatly exaggerated in, large males only, whereas smaller males express greatly reduced or rudimentary horns. On the population level, this results in a bimodal distribution of horn lengths and thus two more or less discrete morphs (Figure 2). Intermediate morphologies do exist, but are rare in most species. As a consequence, populations of conspecific males express a characteristic scaling relationship, or allometry, between body size and horn length (Figure 3). Different species have diversified greatly in the degree of male horn polyphenism and the exact shape of the associated allometry [20], in extreme cases causing alternative conspecific morphs to be classified as different species [21]. 

 Second, smaller males (often referred to as “hornless males” “minor males,” or “sneaker males”) do not invest in horns and fights as means of securing matings, but instead invest in non-aggressive tactics, including the use of enlarged testes and ejaculate volumes to aid in sperm competition [22]. As with horns, morph-specific differences in testes development differ greatly from one species to the other, but comparative studies have not been able to identify any general relationship between the relative sizes of horns and testes [23, 24].

 Third, the facultative enlargement of horns in large males appears to tradeoff with a variety of other structures. The precursors of adult horns develop, just like the precursors of wings, legs, and mouthparts, right before the larval-pupal transition, but *after *all larval feeding has ceased. As such, the development of horns is, like that of all other adult traits, largely enabled by a finite amount of resources accumulated during the larval stage [25]. Structures that develop in the same body location or at the same developmental time may therefore find themselves competing for a limited pool of resources to sustain their growth [26]. When faced with resource allocation tradeoffs, developmental enlargement of one structure may only be possible through the compensatory reduction of another. As such, resource allocation tradeoffs have the potential to not only alter developmental outcomes, but to also bias evolutionary trajectories. In horned beetles, resource allocation tradeoffs have been implicated in antagonistic coevolution of horn length and the relative sizes of eyes, wings [27], and copulatory organs [28], although the exact nature of these tradeoffs remains to be investigated.

### 4.3. Behavioral Plasticity

Alternative horned and hornless male morphs employ different behavioral repertoires to maximize breeding opportunities [12]. In many species, horned males rely exclusively on fighting behaviors including the use of horns as weapons. Body size is the most important determinant of fight outcome, and among similar-sized males, relative horn length predicts fight outcome in most contests (e.g., [14]). Fights can be long, appear energetically expensive, but are rarely injurious (but see [29]). Horned losers typically withdraw from fights.

 Hornless males also engage in prolonged fights when confronted with other hornless males, but quickly withdraw from fights against large, horned conspecifics and switch to a set of non-aggressive sneaking behaviors. For instance, in *Onthophagus taurus*, perhaps the best studied horned beetle species, sneaking behaviors include the use of naturally occurring tunnel interceptions to locate and mate with females without being detected by a guarding male [14]. Small males may also dig their own shallow intercept tunnel to access females underneath guarding males, or wait for females above ground as they emerge periodically to collect dung provisions. Lastly, small males may simply wait next to tunnel entrances for opportunities to temporarily gain access to females while the guarding male is distracted, for instance by fighting off a second intruder. Studies have provided evidence consistent with the hypothesis that hornlessness increases maneuverability inside tunnels, suggesting that the absence of horns may be adaptive in the particular behavioral niche inhabited by small, sneaking males [30].

 Male morphs also differ distinctly in nature and extent of paternal investment. Horned males generally assist females in tunneling and brood ball production, whereas small, hornless males invest most to all of their time into tunnel defense and the securing of additional mating opportunities [31].

 Lastly, behavioral plasticity is not limited to males but also exists in females. Two contexts are especially relevant. First, females typically reproduce by provisioning food for their offspring in the form of brood balls buried underground. In the process, females of at least some species utilize a wide range of dung types and qualities. For instance, *O. taurus* females routinely utilize horse and cow dung in the field. Both dung types differ substantially in quality, and nearly twice as much cow dung than horse dung is needed to rear an adult of similar body size in the laboratory [32]. Individual mothers respond to this variation in dung quality by roughly doubling brood ball masses when offered cow instead of horse dung. Second, females facultatively switch from brood-provisioning behavior to brood-parasitic behavior and the utilization of brood balls constructed by other females [33]. In most cases, a brood-parasitic female will consume the egg inside and either replace it with one of her own while leaving the remainder of the brood ball intact, or incorporate the brood ball into a new, larger brood ball she is constructing herself. Under benign, *ad lib* laboratory breeding conditions up to 13% of brood balls may be affected by such facultative brood-parasitic behavior. This incidence rate roughly doubles when breeding conditions are made adverse by increasing dung desiccation rates [33].

### 4.4. Physiological Plasticity

Recent studies have discovered an unexpected amount of plasticity in thermoregulatory properties and preferences among morphs, sexes, and species of horned beetles. Specifically, Shepherd et al. [34] observed that the ability to be active at high temperatures increased substantially with male and female body size in a species with a modest sexual and male dimorphism. This was also observed in a second species except for large males, which express extremely large thoracic horns, yet exhibited the thermoregulatory behavior of small, hornless males and females. Using these and additional observations, Shepherd et al. [34] suggested that horn development and possession adversely affect the thermoregulatory abilities of male beetles, and that the magnitude of this effect depended on the degree of horn exaggeration. Specifically, they proposed that large, heavily horned males lack the thermoregulatory ability of their large female counterparts, possibly due to a tradeoff between horn production and investment into thoracic musculature, which plays an important role in the shedding of excess heat in scarab thermoregulation [35]. If so, large horned males may be forced to be active at lower temperatures to avoid risking overheating. Preliminary biochemical analyses of thorax protein content are at least partly consistent with such a scenario (Snell-Rood, Innes, and Moczek, unpublished).

 In summary, developmental plasticity pervades the biology of horned beetles, providing rich opportunities to investigate the epigenetic mechanisms underlying plastic responses alongside the ecological and behavioral contexts within which they function and diversify. One genus in particular, *Onthophagus*, has emerged as an especially accessible study system, in large part due to a growing toolbox of developmental genetic and genomic resources. In the next section, we review what we have learned from the application of these tools in the study of the epigenetic regulation of developmental plasticity in these charismatic organisms.

## 5. Epigenetic Mechanisms Underlying Developmental Plasticity in *Onthophagus*

### 5.1. Gene Expression

Microarray applications to *Onthophagus *horned beetle development have been used to quantify and characterize the degree to which the plastic expression of alternative male phenotypes is associated with changes in gene expression [36, 37]. For instance, Snell-Rood et al. [36, 37] used microarrays to examine single-tissue transcriptomes of first-day pupae to contrast male morph-specific gene expression with sex- and tissue-specific gene expression. Several important findings emerged from this work. First, if the same tissue type was examined across alternative morphs (and sexes), transcriptional similarities overall far outweighed differences. Second, for those genes that were significantly differentially expressed across morphs, the frequency and magnitude of differential expression paralleled or exceeded that observed between sexes. In other words, if differential expression is used as a metric of developmental decoupling, the development of alternative morphs appeared just as decoupled as did the development of males and females. Lastly, degree and nature of differential expression varied in interesting ways by tissue type. For instance, the transcriptomes of developing head horns in *O. taurus* were more similar between hornless males and females than to the corresponding tissue region in presumptive horned males. In other words, the head horn transcriptome of small, hornless males appeared feminized, which may not be surprising as both females and small males inhibit horn expression. In contrast to head horns, thoracic horns are enlarged in all* O. taurus* males compared to females but develop transiently, such that they are only visible in pupae yet become resorbed prior to the pupal-adult molt. Transcriptomes of thoracic horns for both male morphs were more similar to each other compared to that of females, and a similar pattern was observed in developing legs. Lastly, brain gene expression patterns of large horned males were more similar to females than to small hornless males. In other words, opposite to the situation for head horns, brain transcriptomes of *horned* males appeared more feminized. Combined, these data demonstrate that the development of alternative male morphs is associated with an appreciable amount of differential gene expression, the nature and magnitude of which differs significantly by tissue type.

Additional array experiments ([38]; Moczek et al. in preparation) and a growing number of candidate gene studies (e.g., [12, 39–44]) have now begun to investigate the possible functional significance of genes that are expressed in a morph-specific (on/off) or morph-biased (up/down) manner. Several important findings have emerged from these studies. First, the development of horns appears to rely, at least in part, on the function of conserved developmental pathways such as the establishment of proximodistal axis through leg gap genes [39], growth regulation through TGF*β*- and insulin-signaling [41, 43, 45], cell-death mediated remodeling during the pupal stage [40], or positioning through Hox- and head gap-genes ([42]; Simonnet and Moczek, unpublished). Second, not all genes expressed during the development of large horns are functionally significant. For instance, the transcription factor *dachshund (dac) *is expressed prominently during the development of both head and thoracic horns, yet RNAi mediated *dac *transcript depletion does not result in any detectable horn phenotypes, despite pronounced phenotypic effects in nonhorn traits [39]. Third, different horn types, whether expressed by different species, sexes, or in different body regions of the same individuals, rely at least partly on different developmental mechanisms and thus may have had different and independent evolutionary histories [46]. Combined, these findings illustrate that the evolution and diversification of horn development have been enabled by the differential recruitment of preexisting developmental mechanisms into new contexts, resulting in a surprising functional diversity within and between species.

### 5.2. Gene Expression—Future Directions

Except for a few well-studied models such as the honey bee [47] or *Daphnia* water fleas [48], little is known about the overall genome-wide magnitude and nature of conditional gene expression. Similarly, we know little about how conditional gene expression compares to other forms of context-dependent gene expression, such as tissue-, stage-, or sex-specific expression. Such comparative data are critical to evaluate whether (a) differential expression of largely similar or different gene-sets underlie different types of context-dependent changes in gene expression; (b) the extent of pleiotropic constraints that might delineate evolution of context-dependent gene expression; (c) the degree to which environment-specific gene expression may result in relaxed selection and mutation accumulation.

Studies on *Onthophagus *beetles have made a first attempt to address a subset of these questions. As detailed above, preliminary array studies identified that the development of alternative, nutritionally cued male morphs is associated with a considerable amount of morph-biased gene expression, the nature and magnitude of which exceeded that of sex-biased gene expression for some tissue but not others, a level of complexity likely to be overlooked by whole-body array comparisons [36]. Furthermore, genes with morph-biased expression were more evolutionarily divergent than those with morph-shared expression, consistent with predictions from population-genetic models of relaxed selection [36, 49, 50] as well as results from other studies (*Drosophila*: [51]; aphids: [52]; bacteria: [53]).

 Additionally, recent work has raised the possibility that conditional gene expression, rather than resulting in relaxed selection, is instead enabled by it. Studies on both Hymenoptera [54] and amphibians [55] show that genes expressed in a morph-biased manner exhibit patterns of sequence evolution consistent with relaxed selection not only in polyphenic taxa, but also related taxa lacking alternative morphs. This suggests that genes exhibiting relaxed selection (for whatever reason) may preferentially be recruited into the expression of alternative phenotypes. If correct this would suggest the possibility for positive feedback, as conditional expression would further relax selection, hence further increasing the probability of recruitment into a plasticity context. Lastly, it is conceivable that the initial relaxation of selection that might enable recruitment of genes for the expression of alternative morphs was facilitated by more subtle forms of plasticity and conditional-gene expression in ancestral, monomorphic taxa, such as season- or sex-biased expression. Ultimately, evaluating the relative significance of the *plasticity-first* versus the *relaxed selection-first* hypotheses (and the potential interplay between them) will require a more thorough sampling of transcriptomes across clades, and most importantly, a more thorough understanding of the developmental functions and fitness consequences of conditional gene expression. Research on *Onthophagus* beetles has the potential to contribute to these efforts through the use of recently developed next-generation transcriptomes and corresponding microarrays [56] as well as studies currently under way to analyze patterns of SNP diversity and sequence evolution within and between species.

### 5.3. Endocrine Regulation

Endocrine mechanisms play a critical and well-established role in the epigenetic regulation of insect plasticity (reviewed in [57, 58]). Findings supporting a role of endocrine factors in the regulation of polyphenism in *Onthophagus* are derived primarily from hormone manipulation experiments, hormone titer profiling, and more recently, gene expression and gene function manipulation studies, as summarized below.

 Juvenile hormone (JH) is a sequiterpenoid hormone secreted by the insect *corpora allata* that maintains the current developmental stage across molts. Applications of a JH analog, methoprene, during *Onthophagus* development provided some of the first evidence that endocrine factors may regulate the expression of alternative nutritionally cued male morphs. Specifically, applications of JH analogs induced ectopic horn expression in *Onthophagus taurus *larvae fated to develop into small, hornless males [59]. In addition, *O. taurus* populations that have diverged in the body size threshold for horn induction showed corresponding changes in the degree and timing of JH sensitivity [60]. Subsequent work on other species has provided additional evidence that JH applications can alter aspects of horn expression, and do so differently for different species, sexes, and horn types [61].

 Ecdysteroids play a critical role in initiating the onset of the molting cycle, and for this class of hormones direct titer measurements do exist for a single *Onthophagus* species, *O. taurus *[59]. Expectedly, ecdysteroid titers were observed to increase in male and female *O. taurus* approaching the larval-pupal molt. However, Emlen and Nijhout [59] also observed a small ecdysteroid peak several days earlier during the feeding phase of the last larval instar. This particular peak in ecdysteroid titers was found in female larvae and male larvae fated to develop into the small, hornless morph, but not in males fated to develop into the large, horned morph. Ecdysteroids have been shown to play a major role in inducing changes in gene expression in developing tissues [62] and Emlen and Nijhout [59] therefore suggested that the low ecdysteroid titers observed in female and small male larvae may facilitate development of a hornless morphology in both groups of individuals via a shared endocrine regulatory process. However, ecdysteroid titers have never been replicated in this or any other *Onthophagus* species, and functional tests using ectopic ecdysteroid applications failed to confirm a function of the early ecdysteroid peak in both females and small males (D.J. Emlen, personal communication).

 Most recently, transcriptional profiling combined with candidate gene studies have provided additional, albeit somewhat indirect support for a role of endocrine regulators during horned beetle development. For instance, Kijimoto et al. [40] investigated the dynamics of programmed cell death during horn remodeling using cell death-specific bioassays. Integrating findings from a companion microarray study, the authors also showed that several genes known to be associated with ecdysteroid signaling in *Drosophila* were expressed in a manner consistent with a role of ecdysteroid signaling in the regulation of horn-specific programmed cell death. Similarly, a combination of candidate gene expression data [45] and array-based transcriptional profiling [37, 40] has begun to implicate signaling via insulin-like growth factors in the regulation of male horn polyphenism. A subsequent functional analysis of *FoxO *[43], a key growth inhibitor in the insulin pathway, has now provided the first functional data in support of such a role (and see below).

### 5.4. Endocrine Regulation—Future Directions

Despite the progress summarized above, our understanding of how endocrine mechanisms influence *Onthophagus* development and behavior lag far behind what is known in other insect model systems, such as photoperiodically cued wing dimorphism in crickets (reviewed in [63–65]) and nutritionally cued caste-development in honey bees (reviewed in [66, 67]). Furthermore, most insights, in particular pertaining to juvenile hormone, have been derived solely from hormone manipulation experiments, whose lack of precision and possible pharmacological side effects limit confidence in the results [63]. While these data are consistent with a functional role of JH in the regulation of developmental plasticity in horned beetles, it is worth noting that direct JH titer profiles have yet to be empirically determined across morphs and sexes for any *Onthophagus* species. Furthermore, direct functional interactions between JH and potential targets relevant for the development of alternative male morphs have yet to demonstrated. Consequently, existing models of JH's role in the development and evolution of horn polyphenism remain largely hypothetical and await critical experimental validation. A recent study by Gotoh et al. [68] is now the first to combine observations of hormone titers with manipulation experiments to demonstrate the role of juvenile hormone in promoting mandible length in a stag beetle, a group of beetles closely related to the Scarabaeidae. These findings motivate complementary studies in horned beetles, which now appear particularly feasible given the recent development of many critical resources.

 Research advances in determining gene function and comparative gene expression have raised the possibility that work in the near future will be able to ascertain more clearly the role of hormones in *Onthophagus* ontogeny, characterize the interplay between genetic and endocrine regulators of development, and examine their respective evolution across species that have diverged in nature and magnitude of developmental plasticity. For example, RNA interference protocols now work routinely and reliably in *Onthophagus* beetles and have already permitted comparative gene function analyses of a variety of key developmental regulators [39, 41, 42, 69], including components of endocrine pathways [43], providing numerous avenues for future research. Furthermore, next-generation transcriptomes [56] of at least two species have massively increased access to relevant sequence information, with additional transcriptomes of other *Onthophagus* species forthcoming.

### 5.5. DNA Methylation

The role of DNA methylation in development and developmental plasticity of *Onthophagus* beetles is still poorly understood, but preliminary evidence suggests that these organisms could be an important system in which to better understand the genetic underpinnings and evolutionary consequences of methylation. First, *O. taurus* has joined the ranks of other emerging insect models, including honeybees, aphids, and parasitic wasps, in containing a complete set of methylation machinery, such as the *de novo* methyltransferase (dnmt3) and the maintenance methyltransferase (dnmt1) [56, 70–72]. Second, a pilot study now suggests that differential methylation is associated with nutritional environment in at least one species, *O. gazella*, and correlated with performance across nutritional environments [73]. This study used a methylation-specific AFLP analysis to survey methylation patterns in family lines derived from a wild population and reared in two different dung types across successive generations. Two major findings emerged. First, methylation state was most heavily influenced by genotype (family line), then rearing environment (dung type), as well as genotype-by-environment interactions (different lines tended to be methylated at different sites when reared on different dung types). Second, methylation state had a significant effect on performance, measured as body size, but in a surprisingly sex- and environment-specific manner: methylation state affected the performance of males (but not females) on cow dung, with the reversed pattern observed on horse dung. Intriguingly, the family line with the greatest flexibility in methylation across environments also showed the highest consistent performance across those environments. Combined, these data are consistent with the hypothesis that facultative methylation underlies adaptive, plastic responses to variation in nutritional environment.

### 5.6. DNA Methylation—Future Directions

The patterns, function, and phenotypic consequences of DNA methylation in insects have received increased attention in recent years, in part for two major reasons. First, insects were once thought to be devoid of methyltransferase enzymes as found in mammals due to the lack of such machinery in the model insect *D. melanogaster*. Subsequent studies have shown that DNA methylation is also absent in two other major invertebrate models, the beetle *T. castaneum* and the nematode *C. elegans* [74]. Phylogenetic reconstructions now suggests rather than reflecting ancestral states, all three lineages have lost aspects of DNA methylation independently [75]. This now provides a unique opportunity to determine the relevance of DNA methylation in development and evolution of phenotypic diversity, plasticity, and integration. Second, genomic methylation patterns and their impact upon transcription in insects are very different from patterns in other taxa. In mammals, genomes are heavily methylated, both in intergenic and intragenic regions, and are generally associated with gene silencing (reviewed in [76, 77]). In many invertebrates, however, genomes appear to be mosaically methylated, with methylation occurring disproportionately in intragenic regions of constitutively expressed housekeeping genes (reviewed in [77]). Thus, studies in emerging and nonmodel insects could allow further understanding of the function of DNA methylation in transcriptional and posttranscriptional regulation [78].

In establishing a correlation between methylation patterns, diet, and performance (body size), the study by Snell-Rood et al. [73] summarized above raised the intriguing possibility that methylation patterns influenced by diet could mediate plastic responses during development in *O. gazella*. If correct, the incredible diversity in nutritional responses that exist within and among *Onthophagus* species would provide a remarkable opportunity to explore the evolutionary diversification of methylation-mediated nutritional plasticity. Such studies would be especially powerful if methylation patterns could be linked to gene regions (e.g., through the use of bisulfite sequencing approaches) and replicated separately for different tissue types, such as gut, epithelium, and brain tissue.

### 5.7. Conditional Crosstalk between Developmental Pathways

The growing number of studies investigating the genetic regulation of horned beetle development has begun to provide the first insights into how different developmental pathways and processes might interact, including facultative interactions depending on nutritional conditions. For instance, Kijimoto et al. [44] investigated the role of *Onthophagus doublesex (dsx)*, a transcription factor known to regulate the sex-specific expression of primary and secondary sexual traits in diverse insects (reviewed in [79]). As in other taxa, *Onthophagus dsx* is alternatively spliced into male- and female-specific isoforms, and consistent with findings from other studies, *male-dsx (mdsx)* and *female-dsx (fdsx)* isoforms promote horn development in male and inhibit it in female *O. taurus*, respectively. Remarkably, *O. taurus mdsx* appears to have evolved the additional function to regulate the development of male horn polyphenism, as evidenced by the following observations. First, *mdsx* is expressed at much higher levels in the head and thoracic horn primordia of large males compared to their legs or abdomen, or when compared to any tissue examined in smaller males. Second, *mdsx*RNA*i* dramatically reduced horn expression in large males only, but left smaller males unaffected. Intriguingly, downregulation of *fdsx* in female *O. taurus* resulted in the nutrition-dependent *induction* of ectopic head horns. Combined, these data suggest that sex- and tissue-specific *dsx* expression and function underlie not only sexual dimorphism, but also male polyphenism in horn expression [44]. The utilization of *dsx* as a regulator of both sexual and male dimorphism may also explain the tight coevolution of both patterns of phenotype expression as reported by earlier phylogenetic studies [20], which found that 19/20 instances of gain or loss in sexual dimorphism were paralleled by a corresponding gain or loss of male dimorphism. Exactly how *dsx* expression and function may be coupled to nutritional input, however, is presently unclear, though several promising candidate mechanisms exist.

 One such candidate is signaling via insulin-like peptides, a pathway well-known for its role in coupling nutritional variation to a wide range of developmental responses, including growth [80]. Differential expression of members of the insulin signaling pathway during facultative horn development have been documented by both a candidate gene study on the insulin receptor [45] as well as array-based transcriptional profiling [37, 40]. The latter studies identified a particularly intriguing member of this pathway, the *forkhead box subgroup O* gene, also known as *FoxO*, as being differentially expressed across several tissue types and nutritional responses. *FoxO* is a growth inhibitor which is typically activated during poor nutritional conditions. Array-based expression evaluations suggested that, relative to abdominal tissue of the same individual, the horn primordia of insipient large males showed much lower *FoxO* expression than the horn primordia of small males, consistent with a role of *FoxO* inhibiting horn growth in small, but not large, males. More detailed qRT-PCR-based expression analyses revealed that contrary to these initial inferences, *FoxO* was not differentially expressed in the horn primordia of large and small male *O. taurus*, but was instead overexpressed in the abdomen of large males, in particular in regions associated with the development of genitalia, including testes. In comparison, the abdomen of small males showed reduced *FoxO* expression. Thus, *FoxO* expression differences in the abdomen of large (high) and small (low) males, rather than expression differences in their horn primordia, accounted for the initial array-based expression data.

 Recall that small males, while reducing investment into horns, invest heavily into genital development, in particular testes mass and ejaculate volumes [23, 81, 82]. Low *FoxO* expression in presumptive testes tissue is consistent with a role of *FoxO* in the upregulation of testicular growth in small males relative to more inhibited growth, marked by elevated *FoxO* expression, in large males. Subsequent RNAi-mediated depletion of *FoxO* transcripts resulted in extended development time and larger body size at eclosing, consistent with a general disinhibition of growth. Moreover, *FoxO-*RNA*i *disrupted the proper scaling of male body size with copulatory organ size, further supporting that *FoxO* may regulate morph-specific genitalia development in horned beetles [43]. In particular, small male genitalia lost their body size dependence whereas large male genitalia exhibited reduced development. Lastly, *FoxO-*RNA*i *modestly but significantly increased the length of horns in large males. Since *FoxO* is *not* differentially expressed in different horn primordia, this finding suggests that elevated horn development observed in large RNAi males might be a secondary consequence of *FoxO*-RNAi-mediated reduction in genitalia development in those same males. More generally, these results raise the possibility that *FoxO* regulates relative growth and integration of nutrition-dependent development of body size, horn length, and genitalia size.

### 5.8. Conditional Crosstalk—Future Directions

How different body parts and tissue types communicate with each other during development, and how their varied scaling relationships are enabled along a continuum of body sizes and in the face of nutritional variation, represent long-standing questions at the interface of developmental and evolutionary biology. Answering these questions is critical to our understanding of the nature of phenotypic integration. Horned beetles are now uniquely positioned as a model taxon in which to identify, on one side, nutrition-responsive developmental pathways and the nature of their interactions with other pathways during development of different body parts and tissues. On the other, the diversity of nutritional responses that exist within and among sexes, populations, and species all provide fantastic substrate for future research efforts into the developmental causes and evolutionary consequences of phenotypic integration.

## 6. Opportunities and Challenges in *Onthophagus* Epigenetics

### 6.1. Stepping Back

Adaptive developmental plasticity allows organisms to modulate their phenotype in response to external environmental cues, permitting developing organisms to better cope with variation in resource availability, physical environment, and social contexts [2]. Plasticity has been of interest to biologists for over a century, and the increased accessibility of molecular data and technology is now enabling an exploration of the molecular underpinnings of this developmentally, ecologically, and evolutionarily central phenomenon [83]. Epigenetic processes have emerged as a diverse and important collection of mechanisms that mediate the interaction between environment and the genome at multiple scales, enabling the expression of developmentally plastic phenotypes (reviewed in [83, 84]). Studies of traditional model organisms have provided powerful insights into the nature and consequences of epigenetic mechanisms. For example, through murine models we have learned that endocrine disruptors, such as the pesticide vinclozolin, can impact not only an exposed individual, but can lead to physiological and behavioral changes in unexposed offspring and grand-offspring. Furthermore, gene knockout lines have subsequently allowed researchers to elucidate some of the molecular underpinnings of this particular phenomenon, mainly epimutations in the germline (reviewed in [85, 86]). Although model organisms are clearly useful for investigating mechanisms underlying epigenetic processes, studies in these organisms have limited power to investigate the relative significance of epigenetics in naturally occurring populations. For instance, many laboratory strains of model organisms are highly inbred, and likely fail to capture the richness of genetic and epigenetic variation found in natural populations [87]. Similarly, one reason that many model organisms were initially selected is that they are phenotypically resilient to variation in the environment, making the study of plasticity in these organisms difficult [87]. New models will thus be important in addressing questions regarding the role of various epigenetic processes in regulating developmental plasticity.

Here, diet-induced plasticity stands out as a particularly important and widespread form of plastic development. Variation in diet quality represents a challenge faced by most, if not all, heterotrophic organisms, and numerous diverse developmental strategies have evolved to cope with diet variation. Moreover, understanding how diet and genes interact during development to form adult phenotypes is essential to understanding how experiences in early life can promote trajectories toward disease later on. Here, we contrast these findings to what is known about the epigenetic control of plasticity in other emerging and established insect models, and close by highlighting several research areas in which future research on *Onthophagus *beetles could potentially contribute to the growing knowledge of the role of epigenetics in regulating developmental plasticity in general and diet-induced plasticity in particular.

### 6.2. The Development and Evolution of Shape

Much variation in organismal shape is the product of evolutionary tinkering in the location, allometry, or function of preexisting structures. Thus, the ultimate factors that promote diversification of shape, as well as the proximate underpinnings that coordinate adaptively proportioned traits, are both of fundamental interest in evolutionary-developmental biology. Adaptive radiations, textbook examples of extensive phenotypic variation stemming from a single ancestral phenotype, have long been used as models to address questions of both ultimate and proximate causes of shape evolution (reviewed in [88]). For instance, the flexible stem hypothesis, pioneered by West-Eberhard [2], suggests that phenotypic diversification observed in adaptive radiations results from selection upon ancestral phenotypes made possible by developmental plasticity. Specifically, ancestral plasticity links the expression of conditional phenotypic variants to particular inducing conditions, thus delineating the nature of phenotypic variation that selection can later act upon in different environments. The flexible stem hypothesis therefore has the potential to explain the common observation of very similar phenotypes arising repeatedly yet independently during adaptive radiations (e.g., [11, 89]). More generally, this hypothesis highlights the potential importance of preexisting plasticity in enabling any kind of evolutionary change, including changes in shape and scaling, by creating the potential for facultatively expressed trait variants to become genetically stabilized and accommodated in descendent generations (see also next section).

*Onthophagus* beetles provide several interesting opportunities to explore the role of plasticity in the diversification of shape and scaling relationships. For instance, adult thoracic horns emerge during development from pupal precursors that originally carried out a very different function [90]. Ancestrally, pupal thoracic horns were resorbed prior to the adult molt, yet descendent species have evolved various ways of partially or fully retaining thoracic horns into adulthood and shaping them into sex- and species-specific weapons. In a subset of species, degree of resorption itself is nutrition dependent [91]. Furthermore, spontaneous retention of thoracic horns also can be observed on occasion in laboratory colonies of species that normally constitutively resorb horns, possibly in response to stressful environmental conditions [40]. This raises the possibility that the diversification of thoracic horn shape and size may have been made possible by harnessing some of the condition dependency of horn retention that existed in ancestral taxa.

A second example involves the well-defined body size thresholds separating alternative horned and hornless male morphs in many species. The exact location of this threshold has diversified greatly among species (Figure 3) as well as some populations. In *O. taurus*, for instance, exotic populations in the Eastern United States, Eastern Australia, and Western Australia have diverged remarkably from their Mediterranean ancestor since introduction approximately 50 years ago [92]. Some of these divergences are similar in magnitude to those observed between well-established species. Intriguingly, body size thresholds are also subject to seasonal or geographic fluctuations in larval nutrition [60, 93] brought about by changes in dung quality and/or changes in the intensity of competition over breeding resources. Again, this raises the possibility that some of the threshold divergences observed between populations and species may have been facilitated initially by conditional responses to altered growth or social conditions.

### 6.3. Evolution via Genetic Accommodation

Genetic accommodation posits that environmental conditions interacting with developmental processes generate phenotypic transformations that can subsequently be stabilized genetically through selection operating on genetic variation in a population. Genetic accommodation does not require new mutations to occur, but will take advantage of them alongside standing genetic variation. Evolution of novel traits and norms of reaction by genetic accommodation have been demonstrated repeatedly and convincingly in artificial selection experiments (reviewed in [5]). Similarly, studies on ancestral plasticity and cases of contemporary evolution provide growing evidence consistent with a role of genetic accommodation in diversification of natural populations (e.g., [11, 94]). However, exactly how important environmental induction really is in the origin and diversification of novel phenotypes remains largely to be determined, in particular in natural populations. Similarly, the proximate mechanisms underlying plasticity-mediated diversification are largely unknown.

 The preceding section highlighted two examples, the diversification of thoracic horn size and shape and the diversification of size thresholds, where research on horned beetles has the potential to generate valuable case studies on the mechanisms and consequences of genetic accommodation of initially environment-induced phenotypic variation. Many additional opportunities exist. For instance, female *Onthophagus* facultatively engage in intra- and possibly interspecific brood parasitism [33]. Interspecific brood parasitism is the dominant reproductive strategy in other dung beetle genera, raising the possibility that it may have evolved initially as a conditional alternative that became subsequently stabilized in a subset of descendent lineages [13]. Similarly, extent of maternal care (brood ball size and depth of burial) vary greatly among females, in part as a function of female body size and thus the nutritional conditions a mother herself experienced when she was a larva. Importantly, *O. taurus* populations obtained from different latitudes within the Eastern US have diverged significantly in the extent of investment mothers provide, again raising the possibility that some of these divergences were enabled initially by plastic responses to environmental conditions (Snell-Rood and Moczek, unpublished data). As highlighted in the last section, *Onthophagus *beetles also provide great opportunities to begin exploring some of the proximate genetic, developmental, and physiological mechanisms that may facilitate accommodation of conditionally expressed phenotypes.

### 6.4. The Origin of Novel Traits

How complex novel traits, such as the eye, the firefly lantern, or the turtle shell, originate is among the most fundamental yet unresolved questions in evolutionary biology [46]. Evolution operates within a framework of descent with modification—anything new and novel must have descended from something old and ancestral. Yet novelties are generally defined as lacking obvious correspondence, or homology, to preexisting traits. How then, do novel traits originate from within the confines of ancestral variation? Studies of epigenetic mechanisms in general, and those focusing on non-model organisms in particular, have likely much to offer to address this question.

 Traditional developmental biology and evo-devo are focused on the identification of genes and gene networks that regulate development and developmental outcomes. At times, this view is expanded to make room for environmental influences by viewing gene function as environment dependent, and viewing genotypes as possessing a reaction norm—that is, the range of phenotypes produced across a range of environmental conditions. The study of epigenetics takes a radically broader and far less gene-centric view. Here, phenotypes (from nucleotide sequences to cells, tissues, organisms, and social groups) emerge as the products of developmental processes to which genes contribute important interactants. In this view, genes are critical and genetic changes can make important differences, but they do not make traits or organisms. Instead, those emerge through the actions of development. This more integrative perspective has many important consequences, three of which are especially critical here. First, epigenetic processes facilitate the production of integrated and functional phenotypes through a wide variety of mechanisms operating well above the sequence level [95, 96]. Second, the integration put in place by epigenetic mechanisms allows development—when confronted with environmental perturbations—to give rise to possibly novel but nevertheless integrated, functional, and on occasion adaptive phenotypes. Third, the same integration enabled by epigenetic mechanisms allows random and modest genetic change to give rise to nonrandom, functional phenotypic changes. In short, the integrity and functionality of phenotypes in development and evolution are facilitated through the chaperoning action of epigenetic mechanisms. As such epigenetics likely plays a central role in facilitating innovation and diversification in nature.

*Onthophagus* beetles have begun to contribute to our understanding of innovation through epigenetic mechanisms through a series of studies focused on the origin and diversification of horns, themselves novel structures lacking any obvious homology to other insect traits (reviewed in [97]). Through a combination of observational, comparative, and manipulation studies it has now become clear that at least some horns originated from pupal-specific structures that originally functioned in completely unrelated contexts (reviewed in [13]). Innovation was enabled initially through the potentially accidental maintenance of normally pupal-specific projections into the adult stage. Similar events can be observed at low frequency in laboratory cultures of species lacking adult horns [40]. Diversification between species, sexes, and morphs was then made possible through the recruitment of preexisting developmental pathways and their targets into a novel context, for instance enabling morph-specific elaboration of horns via preexisting endocrine mechanisms [59, 61, 92, 98] or sex-specific horn expression via sex-specific activation of programmed cell death [40]. Exactly how such recruitment was made possible and by what kind of genetic and environmental variation (and what interactions between them) remain unclear, however, posing some of the many intriguing question for future research in these organisms and the field in general.

## 7. Conclusions

The study of epigenetic mechanisms in development and evolution promises to fill an otherwise abstract genotype-phenotype map with biological reality. Epigenetic mechanisms feature especially prominently in developmental plasticity and its evolutionary consequences. We hope to have shown in this review that the study of horned beetles provides rich and promising opportunities to investigate the role of epigenetics in the evolution of adaptations, phenotypic diversification, and the origin of novel traits. The remarkable degree of plasticity inherent in the biology of horned beetles, combined with the stunning phenotypic diversity that exists both within and among species, and the growing experimental toolbox available for a subset of these organisms makes horned beetles a promising emerging model system in the study of epigenetic mechanisms, their nature, causes, and consequences.
